# Eccentric cycling does not improve cycling performance in amateur cyclists

**DOI:** 10.1371/journal.pone.0208452

**Published:** 2019-01-02

**Authors:** Gøran Paulsen, Hedda Ø. Eidsheim, Christian Helland, Olivier Seynnes, Paul A. Solberg, Bent R. Rønnestad

**Affiliations:** 1 The Norwegian Olympic and Paralympic Committee and Confederation of Sport, Oslo, Norway; 2 Inland Norway University of Applied Sciences, Department of Sport Sciences, Lillehammer, Norway; 3 Norwegian School of Sport Sciences, Department of Physical Performance, Oslo, Norway; 4 The Norwegian Olympic and Paralympic Committee and Confederation of Sport, Region East, Fredrikstad, Norway; Sao Paulo State University - UNESP, BRAZIL

## Abstract

Eccentric cycling training induces muscle hypertrophy and increases joint power output in non-athletes. Moreover, eccentric cycling can be considered a movement-specific type of strength training for cyclists, but it is hitherto unknown if eccentric cycling training can improve cycling performance in trained cyclists. Twenty-three male amateur cyclists were randomized to an eccentric or a concentric cycling training group. The eccentric cycling was performed at a low cadence (~40 revolution per minute) and the intensity was controlled by perceived effort (12–17 on the Borgs scale) during 2 min intervals (repeated 5–8 times). The cadence and perceived effort of the concentric group matched those of the eccentric group. Additionally, after the eccentric or concentric cycling, both groups performed traditionally aerobic intervals with freely chosen cadence in the same session (4–5 x 4–15 min). The participants trained twice a week for 10 weeks. Maximal oxygen uptake (VO_2max_), maximal aerobic power output (W_max_), lactate threshold, isokinetic strength, muscle thickness, pedaling characteristics and cycling performance (6- and 30-sec sprints and a 20-min time trial test) were assessed before and after the intervention period. Inferences about the true value of the effects were evaluated using probabilistic magnitude-based inferences. Eccentric cycling induced muscle hypertrophy (2.3 ± 2.5% more than concentric) and augmented eccentric strength (8.8 ± 5.9% more than concentric), but these small magnitude effects seemed not to transfer into improvements in the physiological assessments or cycling performance. On the contrary, the eccentric training appeared to have limiting or detrimental effects on cycling performance, measured as W_max_ and a 20-min time trial. In conclusion, eccentric cycling training did not improve cycling performance in amateur cyclists. Further research is required to ascertain whether the present findings reflect an actual lack of efficacy, negative effects or a delayed response to eccentric cycling training.

## Introduction

A cyclist’s capacity to release energy and the ability to transfer this energy to pedaling the bike are major performance determinants for cycling. Road cycling performance is primarily limited by aerobic energy capacity, but strength (anaerobic) training has been shown to be a valuable supplementary to the traditional endurance training for cyclists [1,2]. Rønnestad et al [3] showed that 12 weeks of strength training improved cycling economy during long-duration submaximal cycling and increased the mean power output in a final 5 min with maximal effort. Other studies support these findings [4], but the beneficial effects of strength training is not unequivocal [1]. Interestingly, Vikmoen et al [4] found a strong relationship between muscle hypertrophy of the quadriceps muscle and improvements in cycling performance after 11 weeks of strength training (40 min time trial). Moreover, in addition to hypertrophy, the conversion of type IIx to type IIa muscle fibers were also associated with performance improvements [4,5]. Consequently, we could hypothesize that lack of response to strength training in cyclists could be due to a lack of hypertrophy and/or fiber type conversion. It seems reasonable to envision that strength training not always results in muscle morphological adaptations in cyclists, as the strength training is conducted along with large volume of aerobic endurance training [2]. This is consistent with previous reports suggesting that concurrent endurance training may mitigate the effects of resistance training via inhibition of anabolic pathways [6]. We should, however, recognized that strength training induces neural effects, e.g., increased rate of force development (RFD) through increased motoneuron firing frequency [1] and that these neural effects may contribute to better cycling performance independent of muscle hypertrophy [2].

Assuming that strength training-induced hypertrophy and fiber type conversion (IIx to IIa) contribute to improve cycling performance (e.g.: [4]), we can deduce that exercises that activate the IIx motor units (i.e., all motor units) are imperative for cyclists. To this end, the high loading imposed during eccentric exercise has proven effective in inducing muscle hypertrophy and strength, and to initiate type IIx to type IIa conversion [7,8,9,10].

Eccentric cycling was initially introduced as a way to investigate the physiology of concentric and eccentric muscle work [11], and as a model for exercise-induced muscle damage [12]. In recent years, eccentric cycling has been applied to increase knee-extensor strength and hypertrophy in different populations, and to facilitate recovery from injuries, such as anterior cruciate ligament ruptures [9]. Furthermore, Leong et al [13] observed improved maximal concentric cycling power and increased thickness of vastus lateralis and rectus femoris after only 8 weeks of eccentric cycling (5–10.5 min per session) in young, healthy participants (non-athletes). Surprisingly, few studies have included athletes, but Gross et al [14] reported that eccentric cycling (20 min, 3 sessions per week) induced muscle hypertrophy and improved counter-movement jump height in junior alpine skiers. As far as we know, no study has tested the effectiveness of eccentric cycling to improve cycling performance in road cyclists. Furthermore, previous studies have conducted the eccentric cycling on a recumbent bike, while we herein utilized an ordinary bike allowing a more cycling-specific positioning during the exercise.

The principle of specificity has long been documented in relation to the operating range of joints and is believed to be linked to both neural and morphological adaptations [15]. Since conventional cycling requires pure concentric work, the neural adaptations stemming from eccentric cycling is expected to have limited transfer into improved cycling performance [8,16]. However, the specificity of eccentric exercise (lengthening muscle actions at higher force levels) could induce distinct architectural changes of advantage for cycling power output. Based on observations from studies on other forms of eccentric training [17,18], greater regional muscle hypertrophy and longer fascicle length could be expected from eccentric cycling training.

Consequently, the purpose of the present study was to compare the effects of specific eccentric cycling with regular concentric cycling–with the same perceived effort and cadence–on cycling performance and physiological determinants of cycling performance in trained, amateur road cyclists. We hypothesized that eccentric cycling would work as a specific form of strength training and thereby increase knee-extensor thickness (hypertrophy), resulting in improved cycling performance at both short- (anaerobic) and long-duration (aerobic) tests.

## Methods

### Design

The present study was a randomized controlled trial. The participants were randomly allocated to an eccentric cycling group (ECC) or a concentric cycling group (CON). The CON group performed conventional concentric cycling, with the same low cadence and rate of perceived exertion (RPE) as the ECC group (Table 1). In addition to the low cadence eccentric and concentric training, all participants performed traditional aerobic interval training on the same days (Table 1). The participants underwent a 10-week period of supervised training (17 sessions). Performance and physiological tests were conducted within one week before and after the intervention period. The pre-tests were preceded by a test-familiarization session.

**Table 1:** Overview of the intervention period.

Week	ECC/CON	RPE (Borgs scale)	Aerobic intervals 1st session	Aerobe intervals 2nd session	RPE (Borgs scale)
**1**	**Familiarization to tests and pre-testing**				
**2**	5x2 min*	12	4x12 min (83–87% HR_max_)	4x15 min (83–87% HR_max_)*	15–16
**3**	5x2 min	13	4x12 min (83–87% HR_max_)	4x15 min (83–87% HR_max_)	15–16
**4**	6x2 min	13	4x12 min (83–87% HR_max_)	4x15 min (83–87% HR_max_)	15–16
**5**	6x2 min	14	5x8 min (88–92% HR_max_)	4x10 min (88–92% HR_max_)	16
**6**	7x2 min	15–16	5x8 min (88–92% HR_max_)	4x10 min (88–92% HR_max_)	16–17
**7**	8x2 min	15–16	5x8 min (88–92% HR_max_)	4x10 min (88–92% HR_max_)	16–17
**8**	8x2 min	16–17	5x4 min (93–98% HR_max_)	5x6 min (93–98% HR_max_)	17
**9**	7x2 min	16–17	5x4 min (93–98% HR_max_)	5x6 min (93–98% HR_max_)	17–18
**10**	6x2 min	16–17	5x4 min (93–98% HR_max_)	** **	17–18
**11**	**Post-testing**				

*2 min rest periods between all intervals.

CON: Concentric cycling; ECC: Eccentric cycling; HR_max_: Maximal heart rate; RPE: Rate of perceived exertion.

### Participants

The participants were 23 male amateur cyclists (33 ± 12 years and 77 ± 7 kg), with a mean training volume of 10 ± 5 hours per week in the year prior to the study. Within the last 3 months prior to the study, 1.0 ± 1.7 hours per week of strength training had been conducted, and none of the athletes did systematic sprint cycling training (e.g., <30 sec all-out intervals). Based on the criteria presented by De Pauw et al [19], our cyclist could be defined as trained (level 3 or 4 of 5). All cyclists completed the study.

The study was performed according to the ethical standards established by the Helsinki Declaration of 1975 and was approved by the local ethical committee of the Department of Sports Science, Inland Norway University of Applied Sciences, Lillehammer; and The Norwegian Data Protection Authority. All participants signed an informed consent form.

### Cycling tests

All the cycling tests were performed with a Lode Excalibur Sport cycle ergometer (Lode, Groningen, The Netherlands), and conducted in standardized environmental conditions: 16°-18° and 30–40% humidity. The participants were asked to refrain from caffeine and nicotine 4 hours prior to testing and avoid high intensity physical activity the day before testing. Food intake was individually standardized on the test days. The performance and physiological tests were conducted over two days. A blood lactate profile protocol, VO_2max_, and a 20-min time trial were performed during the first day, while the sprint tests were performed along with the isokinetic strength tests on the second day. Twenty minutes of rest was given between the VO_2max_ and the 20-min time trial test, while 10 min was given between other tests.

#### Blood lactate profiling, cycling economy and VO_2max_

With freely chosen cadence, the lactate profile test started at 125 W for 5 min. Thereafter, the load was increased by 50 W every 5 min until reaching a capillary blood lactate concentration ([La^-^]_b_) of 3.0 mmol∙L^-1^. The load was then increase by 25 W until a [La^-^]_b_ of 4.0 mmol∙L^-1^ was reached (Biosen C-line Clinic, EKF Diagnostics, GmbH, Barleben, Germany). The lactate threshold was determined as the power output at 4 mmol∙L^-1^ [La^-^]_b_, calculated from the relationship between [La^-^]_b_ and power output using linear regression between the nearest [La^-^]_b_ below and above 4 mmol∙L^-1^. Cycling economy (W∙ml O_2_^-1^) was calculated from the average oxygen consumption between 3 and 4.5 min of the two first submaximal stages (125 W and 175 W).

The VO_2max_ test was initiated with 1 min of cycling at a power output corresponding to 3 W·kg^–1^ (rounded down to the nearest 50 W). Power output was subsequently increased by 25 W every minute until exhaustion. VO_2max_ was determined by the average of the two highest VO_2_ measurements (30 sec periods), and maximal aerobic power output (W_max_) was calculated as the mean power output of the last minute of the test.

#### Pedal force measurements

The torque generated at the crank axle was measured every 2° by strain gauges developed and bonded on to the crank arm by the Lode cycle ergometer manufacturer. Peak torque, angle of peak torque, and minimum torque were averaged from both legs. Peak torque was calculated as the mean of the highest propulsive torque during the down-stroke phase, while minimum torque was calculated as the mean of the highest resistive torque during the upstroke phase. Crank angles were referenced to 0° at the top dead center and 180° at the bottom dead center; zero adjustment calibration in Lode software was performed prior to every test (Lode Ergometry Manager 9.3.1.0). The crank torque data was recorded as the average from 1.5 to 4.5 min during the 5-min period closest to 4 mmol∙L^-1^ lactate during the blood lactate profile test (273 ± 23 W for the ECC group and 239 ± 42 W for the CON group). At the post-test, individual crank torque measurements were performed at the same power output and using the same cadence as during the pre-test.

#### 20-min time trial

In the 20-min time trial test, the participants aimed for a highest possible mean power output (Lode Excalibur Sport cycle ergometer). The cadence was freely chosen, and the participants controlled the power output during the whole test by using an electronic control unit that governed the electromagnetic brake on the drive wheel of the cycle ergometer (hyperbolic mode). [La^-^]_b_ was measured every 5 min. The amount of water or sports drink consumed were noted during the pre-test and replicated during the post-test.

#### Sprints cycling tests

A 10-min cycle specific warm-up, including two submaximal sprints and 1-min rest, were performed before the 6-sec and 30-sec (Wingate) sprint tests (Lode Excalibur Sport cycle ergometer). The 6-sec test was performed with maximal effort from a standstill (2 attempts; 2 min rest; the best attempt was used for statistical analysis). The 30-sec all-out Wingate test started while pedaling at 60 revolution per minute (RPM) without braking resistance. Then, following a 3-sec countdown, braking resistance was applied to the flywheel and remained constant throughout the test. Braking resistance was set to 0.75 Nm·kg^-1^ body mass on both the 6-sec and 30-sec tests. The cadence was sampled at 5 Hz and matching power output values were calculated (Lode Ergometry Manager 9.3.1.0). The mean power output was presented as the average power output sustained during the 6-sec and 30-sec tests. The cyclists remained seated throughout the tests and strong verbal encouragement was provided. The participants were instructed to pedal as fast as possible from the start and not to conserve energy for the last part of the test (to avoid pacing during the Wingate test).

### Isokinetic strength tests

Seated with 85° in the hips and upper body and thighs stabilized by belts and Velcro bands, the participants were subjected to isokinetic knee-extensor strength tests of their dominant leg (HUMAC NORM, Computer Sports Medicine Inc, Massachusetts, USA). Preceded by 5 warm-up contractions for each velocity, maximal concentric and eccentric strength was tested at 60°·sec^-1^. The highest torque of three consecutive attempts at each velocity was used in further statistical analysis. Maximal knee-extensor isometric torque was assessed at 60° for 5 secs (2 attempts; the highest value used for statistical analysis). One minute and 30 secs of rest was given between tests and warm-ups, respectively.

### Muscle thickness

The ultrasound assessment was always conducted before any other tests and with a minimum of 24 hours rest from the last training session.

Muscle thickness of m. vastus lateralis (VL) and m. rectus femoris (RF) were measured from ultrasound scans (HL9.0/60/128Z-2, Telemed Ltd Lithuania, Echo Wave II, Italy, Milano). Images were obtained at the mid-distance between the greater trochanter and the femoral condyle. Scanning sites were recorded on acetate paper for subsequent measurements. However, great care was taken to match pre- and post-intervention scanning sites by adjusting the probe orientation to display similar landmarks (e.g. connective tissue and blood vessels). All images were analyzed in a blinded fashion by the same investigator using ImageJ (Wayne Rasband, National Institutes of Health, Bethesda, MD). The distance between the superficial and deeper aponeuroses was measured at three different sites in the middle third of the width of the field of view. An average of these measurements was used as muscle thickness.

Ultrasound measurements of muscle architecture have consistently been shown to be valid [20]. The reliability of repeated measurements using the present method has been estimated as acceptable, with coefficient of variation of 2.0% [21].

### Training

#### Eccentric cycling or concentric cycling

Each session started with a 10-min warm-up at low intensity (120–160 W). Table 1 provides an outline of the eccentric and concentric training, which started 2 x 2 min and progressed to 8 x 2 min. Inter-interval rests were always 2 min. The participants in the ECC group performed their training on a Cyclus2 Eccentric Trainer (RBM elektronik-automation GmbH, Leipzig, Germany), while those in the CON group used a Body Bike Classic (BODY BIKE international A/S, Frederikshavn, Denmark). Rate of perceived exertion (Borgs scale 6–20) was used to target intensity during eccentric and concentric cycling (Table 1). Both the ECC and the CON group cycled at a cadence of 40 RPM. The eccentric ergometer displayed the cadence, while the CON group followed the beat of a metronome. Consequently, intensity was individually adjusted with the resistance. Mean force (N), power (W) and heart rate were recorded during the eccentric cycling, while only heart rate was recorded from the concentric cycling.

#### Aerobic intervals

The last part of the training session was equal for all the participants and started with 10 min progressive warm-up to prepare for aerobic intervals (Table 1). During these aerobic intervals, most participants used their own bike on CompuTrainers (RacerMate Inc, Seattle, Washington, USA) with cadence and power output registration, while the remaining cyclists completed the aerobic intervals on a Body Bike Classic. At the end of each session, the participants received a 29 g protein bar (“Big100 bar”, Proteinfabrikken, Stokke, Norway) for recovery purposes.

#### Tapering

The last organized training session and the post-testing were separated by 5 days. In this period, the participants were instructed to perform a step taper by decreasing their individual training volume by 50 percent [22], and to perform a training session of 5 x 2 min aerobic intervals at 93–98% of maximal heart rate and 17–18 on the Borgs scale, two days before post-testing. The purpose with the taper was to allow for recovery and final adaptations before the post-tests [23]

### Statistics

The data were analyzed in a spreadsheet designed for a controlled trial that allows for adjustment of two predictor variables [24]. In all analyses, the differences in changes between groups were adjusted for baseline level to correct for the regression to the mean effect (those with a high score at the pre-test tends to get weaker and those with a low score at the pre-test tends to get better). In addition, the spreadsheet allows for including an additional explanatory variable, and we included changes in VO_2max_ and hypertrophy as possible mediators explaining the differences between training groups. All data were log-transformed and differences between groups are reported as percent with its associated 90% confidence interval (CI).

Effects were evaluated using the clinical magnitude-based inferences (MBI; [25]), a method particularly recommended for small samples. The magnitude of a difference in mean between groups was assessed by standardization, i.e., the mean change divided by baseline standard deviations (SD) of all subjects. The resulting standardized effect was evaluated as following: <0.2, trivial; 0.2–0.6, small; 0.6–1.2, moderate; >1.2, large [25].

To make clinical inferences about true values of effects in the population studied, the effects were expressed as probabilities of harm or benefit in relation to the smallest substantial effect (0.2 SD; [25]). The ratio of wanting to use the experimental training corresponds to the case of an effect that is almost certainly not harmful (<0.5% risk of harm) and possibly beneficial (>25% chance of benefit). This corresponds to an odds ratio of 66 which is according to [24,25] enough to warrant to use the treatment. The effect is shown as the difference or change with the greatest probability, and the probability is shown qualitatively using the following scale: 25–75%, possibly; 75–95%, likely; 95–99.5%, very likely; >99.5%, most likely [25].

Pearson´s correlations between the change in the possible mediators (delta hypertrophy and VO_2max_) and performance tests were performed among all subjects. According to [25] a correlation <0.1 is considered trivial, 0.1–0.3 small, 0.3–0.5 moderate and >0.5 large, and their inferences were evaluated using the same scale as described for effects above.

## Results

Eccentric cycling was based on the perceived effort (Table 1), which resulted in a resistance during each 2-min interval of 400 ± 80 N (290 ± 60 W) during the three first sessions and 700 ± 90 N (520 ± 70 W) during the last three sessions. The heart rate was 115 ± 15 beats per minute (BPM) and 130 ± 15 BPM during the first and last three sessions, respectively. For the CON group, the heart rate was 140 ± 20 BPM during the three first sessions and 150 ± 15 BPM during the last three sessions.

Table 2 shows the mean and SD of all variables in the two groups at baseline. We did not investigate the difference between groups at baseline, because all analyses included baseline as a covariate (controlling for possible differences). Baseline values together with delta changes within each group are presented in S1 Table.

**Table 2:** Descriptive statistics for the main variables in each group at baseline.

	ECC Mean ± SD (n = 12)	CON Mean ± SD (n = 11)
**Muscle thickness**		
Vastus lateralis (VL; mm)	2.7 ± 0.4	2.6 ± 0.3
Rectus femoris (RF; mm)	2.0 ± 0.3	1.8 ± 0.2
Mean of RF and VL (mm)	2.3 ± 0.3	2.2 ± 0.2
**Strength**		
Eccentric peak torque at 60°·s^-1^ (Nm)	241 ± 43	265 ± 52
Eccentric work at 60°·s^-1^ (J)	317 ± 72	317 ± 66
Eccentric power at 60°·s^-1^ (W)	150 ± 28	160 ± 28
Eccentric angle at peak torque at 60°·s^-1^ (°)	79.8 ± 11.8	72.9 ± 11.4
Concentric peak torque at 60°·s^-1^ (Nm)	221 ± 37	229 ± 33
Concentric work at 60°·s^-1^ (J)	278 ± 51	280 ± 60
Concentric power at 60°·s^-1^ (W)	146 ± 25	154 ± 23
Concentric angle at peak torque (°)	63.4 ± 8.1	64.1 ± 7.1
Isometric peak torque at 60° (Nm)	242 ± 41	263 ± 38
**Performance tests**		
6-sec sprint mean power (W)	1276 ± 102	1251 ± 84
30-sec sprint mean power (W)	776 ± 62	761 ± 52
20-min time trial (W)	268 ± 32	260 ± 42
20-min time trial (W·kg^-1^)	3.6 ± 0.6	3.4 ± 0.7
20-min time trial average lactate (mmol·L^-1^)	8.5 ± 1.7	6.6 ± 1.5
**Endurance determinants**		
VO_2max_ (ml)	4668 ± 616	4796 ± 518
VO_2max_ (ml·kg^-1^)	62.1 ± 10.1	62.0 ± 9.4
W_max_ (W)	406 ± 54	383 ± 36
4 mmol·L^-1^ lactate threshold (W·kg^-1^)	3.3 ± 0.7	3.0 ± 0.8
Cycling economy (W·ml^-1^)	16.3 ± 0.9	16.9 ± 0.8
**Pedaling characteristics**		
Pedaling peak torque (N)	69.5 ± 7.2	69.5 ± 9.8
Pedaling efficiency (%)	86.4 ± 3.1	80.6 ± 8.1
Pedaling average angle (°)	90.2 ± 5.6	90.3 ± 5.2
Pedaling min torque (N)	-9.5 ± 1.3	-12.8 ± 3.9
Pedaling cadence (RPM)	94.4 ± 5.6	91.0 ± 4.8
Training volume last 12 weeks (hours per week)	10 ± 4	8 ± 4

CON: Concentric cycling; ECC: Eccentric cycling; RPM: Revolutions per minute.

Table 3 presents the percent difference in mean changes between CON and ECC. The left column shows the effects of the ECC compared to CON when only adjusting for baseline. In general, the differences between groups were trivial or small. There was a small likely beneficial effect of ECC on eccentric strength compared to CON (isokinetic eccentric force/work/power). Moreover, there was a small possibly clear effect on change in hypertrophy in ECC. For the performance tests, the effects of ECC were overall negative small or trivial, with clear negative effects on the 20-min time trial, W_max_ and average pedaling peak torque and angle.

**Table 3:** Percent difference in changes between groups with magnitude-based inferences when adjusted to baseline, adjusted to baseline and change in VO_2max_, or adjusted to baseline and change in muscle volume.

	Diff ECC-CON adjusted for baseline		Diff ECC-CON adj for baseline and delta VO_2max_		Diff ECC-CON adj for baseline and delta hypertrophy	
	Mean diff ± 90% CI	Inference	Mean diff ± 90% CI	Inference	Mean diff ± 90% CI	Inference
**Muscle thickness**						
Vastus lateralis (VL; mm)	2.6 ± 5.2	small^unclear^	4.8 ± 6.7	small^unclear^		
Rectus femoris (RF; mm)	2.6 ± 3.8	small^unclear^	5.5 ± 6.1	small^unclear^		
Mean of RF and VL (mm)	2.3 ± 2.5	small*	4.3 ± 3.4	small**		
**Strength**						
Eccentric peak torque at 60°·s^-1^ (Nm)	8.8 ± 5.9	small**	11.6 ± 9.4	small**	12.6 ± 9.9	small**
Eccentric work at 60°·s^-1^ (J)	5.4 ± 7.1	small^unclear^	7.7 ± 10.5	small^unclear^	16.9 ± 8.9	mod***
Eccentric power at 60°·s^-1^ (W)	8.2°· 10.2	small^unclear^	9.2 ± 13.5	small^unclear^	35.8 ± 13.6	large****
Eccentric angle at peak torque at 60°·s^-1^ (°)	-2.7 ± 6.8	triv^+^	-0.4 ± 11.2	triv^unclear^	-8.7 ± 12.0	small^++^
Concentric peak torque at 60°·s^-1^ (Nm)	-3.1 ± 6.2	triv^+^	-3.8 ± 9.5	small^+^	-9.0 ± 7.5	small^++^
Concentric work at 60°·s^-1^ (J)	-2.5 ± 5.9	triv^+^	-4.5 ± 7.3	small^+^	-5.8 ± 8.5	small^+^
Concentric power at 60°·s^-1^ (W)	-1.9 ± 5.8	triv^+^	-2.8 ± 7.7	triv^+^	-5.3 ± 9.1	small^+^
Concentric angle at peak torque (°)	-0.7 ± 5.6	triv^+^	5.4 ± 7.1	small^unclear^	0.2 ± 8.4	triv^unclear^
Isometric peak torque at 60° (Nm)	3.2 ± 8.9	triv^unclear^	13.4 ± 12.6	mod^unclear^	8.1 ± 15.0	small^unclear^
**Performance tests**						
6-sec sprint mean power (W)	0.9 ± 5.0	triv^unclear^	0.9 ± 6.1	triv^unclear^	3.6 ± 8.5	small^unclear^
30-sec sprint mean power (W)	-0.6 ± 2.5	triv^+^	1.9 ± 3.8	small^unclear^	-0.3 ± 3.1	triv^0^
20-min time trial (W)	-3.5 ± 3.8	small^+^	-3.2 ± 6.4	small^+^	-4.2 ± 4.6	small^+^
20-min time trial (W·kg^-1^)	-2.3 ± 3.9	triv^00^	-2.5 ± 6.7	triv^+^	-3.2 ± 4.8	triv^+^
20-min time trial average lactate (mmol·L^-1^)	3.3 ± 22.1	triv^unclear^	-7.8 ± 26.2	small^+^	-1.0 ± 24.3	triv^unclear^
**Endurance determinants**						
VO_2max_ (ml)	-1.6 ± 2.9	triv^+^			-0.7 ± 3.8	triv^0^
VO_2max_ (ml·kg^-1^)	0.0 ± 3.2	triv^00^			2.8 ± 4.1	triv^unclear^
W_max_ (W)	-3.0 ± 2.9	small^+^			-1.7 ± 3.3	triv^+^
4 mmol·L^-1^ lactate threshold (W·kg^-1^)	-0.9 ± 5.0	triv^00^	-0.3 ± 8.9	triv^0^	0.7 ± 5.3	triv^00^
Cycling economy (W·ml^-1^)	0.3 ± 3.8	triv^unclear^	0.1 ± 5.1	triv^unclear^	-0.9 ± 5.1	triv^+^
**Pedaling characteristics**						
Pedaling peak torque (N)	-5.9 ± 8.2	small^++^	2.9 ± 12.4	small^unclear^	-6.7 ± 9.5	small^++^
Pedaling efficiency (%)	-1.1 ± 3.5	triv^+^	0.6 ± 4.6	triv^unclear^	-0.7 ± 3.7	triv^+^
Pedaling average angle (°)	-2.7 ± 3.4	small^++^	-1.7 ± 5.2	small^+^	-1.8 ± 4.5	small^+^
Pedaling min torque (N)	1.1 ± 18.7	triv^unclear^	-7.7 ± 25.6	small^+^	-3.4 ± 18.0	triv^+^
Pedaling cadence (RPM)	0.4 ± 3.9	triv^unclear^	-4.2 ± 4.9	mod^++^	-0.8 ± 4.5	triv^+^

Magnitude thresholds (for mean change divided by baseline SD of the total sample): <0.20, trivial; 0.20–0.59, small; 0.60–1.19, moderate; >1.20, large.

Asterisks indicate effects clear at the 5% level and likelihood that the true effect is substantial or trivial, as follows

*possible

**likely

***very likely

****most likely.

^+^possibly harmful

^++^likely harmful.

^0^possibly trivial

^00^likely trivial.

CI: Confidence intervals; CON: Concentric cycling; ECC: Eccentric cycling; RPM: Revolutions per minute.

The middle column (Table 3) shows the difference in means when adjusting for baseline and change in VO_2max_, meaning that the delta VO_2max_ between groups is held constant (adjusted to zero). This approach showed overall similar results as adjusting for baseline, but concentric strength (peak torque and work), average blood lactate during 20-min time trial, pedaling minimum torque and pedaling RPM became small and clearly negative for ECC.

The right column (Table 3) shows the effect of ECC (compared to CON) when adjusted for baseline and hypertrophy (mean of VL and RF). The differences in the performance tests were still negative and trivial or small (20-min all-out W, possibly harmful). If anything, the differences between ECC and CON on the performance tests became larger when controlling for the change in hypertrophy indicating that the effect of hypertrophy on cycling performance was negative for the ECC group.

The effects of change in the possible mediators, VO_2max_ and hypertrophy, on the differences between the two groups are given in Table 4. Generally, the effects of the mechanisms were trivial or small and unclear. However, for change in VO_2max_ there were some clear negative effects of VL muscle thickness, 30-sec sprint mean power, pedaling peak torque, pedaling mechanical efficiency, concentric angle of peak torque and isometric peak torque, indicating that the increases in VO_2max_ in ECC negatively influenced these outcomes compared to CON.

**Table 4:** Percent effect of change in VO_2max_ and change in hypertrophy (mediators) on the difference between groups.

	Effect of delta VO_2max_		Effect of delta hypertrophy	
	Mean diff ± 90% CI	Inference	Mean diff ± 90% CI	Inference
**Muscle thickness**				
Vastus lateralis (VL; mm)	-1.4 ± 4.2	triv^+^		
Rectus femoris (RF; mm)	-2.7 ± 3.7	small^+^		
Mean of RF and VL (mm)	-1.6 ± 2.0	triv^+^		
**Strength**				
Eccentric peak torque at 60°·s^-1^ (Nm)	-2.5 ± 5.9	triv^+^	-1.8 ± 4.8	triv^00^
Eccentric work at 60°·s^-1^ (J)	-1.9 ± 6.4	triv^0^	-7.2 ± 4.5	small^++^
Eccentric power at 60°·s^-1^ (W)	-0.5 ±8.0	triv^0^	-13.6 ± 5.1	mod^+++^
Eccentric angle at peak torque at 60°·s^-1^ (°)	-2.3 ± 8.1	triv^+^	4.7 ± 7.6	small^unclear^
Concentric peak torque at 60°·s^-1^ (Nm)	0.2 ± 6.8	triv^0^	5.8 ± 5.6	small**
Concentric work at 60°·s^-1^ (J)	1.8 ± 4.9	triv^00^	3.1 ± 5.5	triv^unclear^
Concentric power at 60°·s^-1^ (W)	0.4 ± 5.2	triv^00^	3.1 ± 5.9	triv^unclear^
Concentric angle at peak torque (°)	-5.1 ± 4.1	small^++^	-1.4 ± 5.0	triv^+^
Isometric peak torque at 60° (Nm)	-9.0 ± 7.1	small^++^	-3.4 ± 7.8	triv^+^
**Performance tests**				
6-sec sprint mean power (W)	0.8 ± 3.7	triv^unclear^	-2.5 ± 4.8	small^+^
30-sec sprint mean power (W)	-1.5 ± 2.3	small^+^	-1.3 ± 3.1	triv^+^
20-min time trial (W)	-0.4 ± 5.2	triv^0^	-1.0 ± 2.4	triv^00^
20-min time trial (W·kg^-1^)	0.2 ± 5.9	triv^0^	-1.1 ± 2.8	triv^00^
20-min time trial average lactate (mmol·L^-1^)	11.3 ± 20.6	small^unclear^	8.3 ± 13.2	small^+^
**Endurance determinants**				
VO_2max_ (ml)			-1.4 ± 2.1	triv^00^
VO_2max_ (ml·kg^-1^)			-2.5 ± 2.4	triv^+^
W_max_ (W)			-2.3 ± 1.8	triv^+^
4 mmol·L^-1^ lactate threshold (W·kg^-1^)	-0.6 ± 6.8	triv^00^	-4.5 ± 3.2	triv^+^
Cycling economy (W·ml^-1^)	0.2 ± 3.0	triv^unclear^	1.1 ± 2.3	triv^unclear^
**Pedaling characteristics**				
Pedaling peak torque (N)	-8.2 ± 7.5	mod^++^	-0.3 ± 4.9	triv^0^
Pedaling efficiency (%)	-1.8 ± 3.2	small^+^	-0.6 ± 1.6	triv^00^
Pedaling average angle (°)	-1.1 ± 3.7	triv^+^	-0.6 ± 2.3	triv^+^
Pedaling min torque (N)	9.4 ± 22.4	small^unclaer^	5.7 ± 8.1	triv^unclear^
Pedaling cadence (RPM)	3.9 ± 3.2	mod^unclear^	0.9 ± 1.9	triv^unclear^

Magnitude thresholds (for mean change divided by baseline SD of the total sample): <0.20, trivial; 0.20–0.59, small; 0.60–1.19, moderate; >1.20, large.

Asterisks indicate effects clear at the 5% level and likelihood that the true effect is substantial or trivial, as follows

**likely.

^+^possibly harmful

^++^likely harmful

^+++^very likely harmful.

^0^possibly trivial

^00^likely trivial.

CI: Confidence intervals; CON: Concentric cycling; ECC: Eccentric cycling; RPM: Revolutions per minute.

For hypertrophy, there were some clear negative effects on 6-sec sprint mean power, eccentric work and power indicating that increased hypertrophy had negative effects in ECC compared to CON (Table 4)

Including all participants (n = 23), there were large clear correlations between change in hypertrophy (RF+VL) and change in W_max_ (0.62, most likely positive) and change in W·kg^-1^ at 4 mmol·L^-1^ [La^-^]_b_ (0.56, very likely positive), and a moderate correlation to average W·kg^-1^ during the 20-min time trial out (0.48, very likely positive).

## Discussion

In the present study, we hypothesized that low cadence eccentric cycling would induce more muscle hypertrophy in the knee-extensors than perceived effort-matched low cadence concentric cycling, and that this muscle hypertrophy would translate into improved cycling performances in amateur cyclists. The main findings were 1) eccentric cycling induced hypertrophy of vastus lateralis and rectus femoris, 2) eccentric cycling resulted in improved isokinetic eccentric strength, which did not transfer to isokinetic concentric strength nor to cycling sprint performance, 3) eccentric cycling changed the pedaling characteristics by an earlier and lower peak torque during the pedaling stroke, and 4) eccentric cycling demonstrated possible unfavorable effects on W_max_ and the 20-min time trial performance.

### Eccentric cycling and hypertrophy

Eccentric exercise has for years been advocated for inducing hypertrophy and strength [9,26,27], and eccentric cycling appear a viable mode of exercise for this purpose [10,28,29]. In the present study, we noted increased thickness of vastus lateralis and rectus femoris. However, the hypertrophy was (with unclear to likely likelihood) of small magnitude compared to other studies investigating resistance exercise in general (~3% in the present study *vs*. 6–9% as summarized by Wernbom et al [30]). Rønnestad et al [31] reported a quadriceps hypertrophy translating into 0.05% increase in cross-sectional area (CSA) per day in well-trained cyclist, which is close to 0.04% in muscle thickness in the present study (assuming changes in thickness and CSA are comparable [30,32,33]). However, when comparing our results to pure eccentric training regimes, our observations are within the range (0.03–0.09%) of observations summarized by Wernbom et al [30]. Intriguingly, Leong et al [13] reported a very large muscle hypertrophy of vastus lateralis and rectus femoris (13 and 24%, respectively) after only 8 weeks of eccentric cycling (i.e., 0.2–0.4% increase per day). This discrepancy may be ascribed to the untrained status of the participants recruited by Leong et al, in contrast to the trained cyclists of the present study. In fact, our participants appeared to have larger muscle thickness of vastus lateralis at baseline than the participants in the study of Leong et al (~27 *vs*. ~20 mm, respectively); and therefore attenuated hypertrophy could be expected [15]. Years with road cycling may indeed induce muscle hypertrophy *per se* [34].

The reasons for modest effects on muscle thickness in our cyclists after eccentric cycling are not possible to ascertain with the design and methods applied, but some likely culprits are worth mentioning. First, the eccentric exercise was conducted on an ordinary bike, which meant that the athlete had to use considerable upper body force to maintain a seated cycling position during exercise. In contrast, when using a recumbent bike set-up, typically used for eccentric cycling (see: [13,14,35]), the stabilization gained from back support makes it easier to generate eccentric force against the pedals. In other words, the muscle hypertrophy in the present study was possibly limited by a suboptimal loading stimulus. Moreover, eccentric exercise sessions were immediately followed by an aerobic cycling session. During concurrent training, it is well established that aerobic exercise can hinder the hypertrophy response to resistance exercise, especially when aerobic and resistance exercises are conducted with little rest in-between, as in the present study [6,36]. Interestingly, the statistical analyses with changes in VO_2max_ as a mediator revealed that VO_2max_ increased on the expense of vastus lateralis muscle thickness, pedaling peak torque and mechanical efficacy. We combined the training modes in order to fit the intervention into the participants training regimes; however, better results could probably have been achieved by separating the eccentric training from the aerobic endurance training, and/or applying a block periodization approach [37].

### Eccentric cycling and cycling performance

According to our hypothesis, we observed hypertrophy after eccentric cycling, but no benefits was observed in the cycling tests. It is possible that the hypertrophy response was too small to result in performance enhancements. Vikmoen et al [4] observed a ~7% CSA quadriceps enlargement (*vs*. ~3% in the present study) and strong correlation with 40 min performance test (mean watts; r = 0.7). Interestingly, when groups were pooled, changes in hypertrophy showed moderate to large correlations to changes in W_max_, W·kg^-1^ at 4 mmol·L^-1^ [La^-^]_b_, and average W·kg^-1^ during the 20-min time trial, indicating that hypertrophy is a mechanism behind the improved cycling performance. It is tempting to speculate whether the eccentric training induced very contraction-specific effects, which somehow blunted or held back the participants ability to utilize the increased muscle mass in conventional cycling tests. In agreement with this suggestion, previous studies have resulted in no or only small improvements in concentric strength/power after 4–8 weeks (2–3 sessions per week) of eccentric cycling [13,35,38]. Leong et al [13] reported robust hypertrophy, but saw only minor transfer to concentric cycling power output one week after the eccentric cycling. Intriguingly, Leong et al [13] reported signs of a delayed response with a larger increase in concentric cycling power 8 weeks after the training intervention. A delayed effect could be related to prolonged muscle remodeling/adaptation process [13,39], and/or time needed to “calibrate” the neuromuscular system to the gained muscle mass. Unfortunately, we were not able to test our cyclists at a later time point (e.g., 2–4 weeks) after the intervention, so we can only hypothesize about a delayed positive effect of eccentric cycling. Of note, we did reduce the training volume in the two lasts weeks and included a 5-day tapering period after the last eccentric training session.

### Specificity of training and possible detrimental effects on performance

The specificity of strength improvements is well-documented [15], and our result confirm an isolated improvement in eccentric strength and power to eccentric training (e.g., [8,16]. Applying changes in muscle thickness as a statistical mediator showed that hypertrophy appeared to limit the improvements in eccentric strength while facilitate concentric strength. This suggests that the eccentric strength increase was brought about via neural adaptions.

Surprisingly, the eccentric training appeared to induce some limiting and even detrimental effects on both isolated concentric strength/power tests, as well as for the W_max_ and the 20-min time trial performances. The adverse effects on the cycling tests may be related to the fact that the eccentric training affected pedaling characteristics, i.e., an earlier and lower peak torque during the pedaling stroke, compared to the concentric group. The group difference in changes of mechanical efficiency was trivial, but also this variable tipped in disfavor for the eccentric training. Confusingly, earlier pedaling peak torque have been observed after traditional strength training and was positively associated with improved 40-min cycling performance [39]. Thus, our observations indicate that the changes in peak torque angle *per se* have limited effect on performance, at least when peak torque is reduced.

Related to high mechanical forces and low metabolic challenges, eccentric exercise may preferentially stimulate type II fibers hypertrophy and in some cases even increase IIx expression [40]. These adaptations could prove counter-productive for aerobic endurance and may explain why we did not find any beneficial effects of tests taxing the aerobic systems. On the other hand, if the eccentric cycling did stimulate type II fibers to grow concomitant with increased IIx fiber type expression, it could be argued that we should have seen improvements in the cycling sprint tests, which we did not. However, a more dominating fiber type II pool may have shifted the optimal cadence for peak power to the right [41], and thus masked true changes in sprint peak power as we tested our cyclists with a single (and similar) load before and after the intervention period.

### Study limitations

The present study lasted for merely 10 weeks and, as previously mentioned, the low to moderate eccentric forces and concurrent training may have restricted the hypertrophy we aimed for. Moreover, we compared eccentric cycling training to concentric cycling training, which means the control group performed more aerobic endurance training than the intervention group. We did not measure oxygen uptake during the eccentric cycling training, but we know from extensive previous work that the cardiovascular load during eccentric cycling is low, even at very high workloads [11,26,42]. Thus, the lower aerobic endurance training load in the intervention group could explain some of the apparently adverse effects of the eccentric cycling. Finally, our results must be interpreted with caution due to the small sample size and many inferences. Some effects were substantial (small), but unclear, indicating that a larger sample size was needed.

### Practical applications and further research

Despite some weaknesses, our study questions the use of eccentric cycling in cyclists as we observed likelihood of adverse effects in cycling performance; which is in contrast to traditional strength training [2]. However, as eccentric cycling seems to have many potential benefits used in both clinical and sport settings [9], it is important to further investigate this mode of training for each specific population. Concerning cyclists, it would be very interesting to test the effects of eccentric cycling in adjunction with traditional strength training exercises, such as leg press and squats, and/or conventional sprint cycling exercise (e.g., 5–30 sec all-out intervals). In this context, it seems reasonable to suggest that the benefits of the hypertrophic stimulus and specific joint movements would give a better transfer to cycling performance.

## Conclusion

Herein we compared low cadence eccentric cycling to perceived effort-matched low cadence concentric cycling training for 10 weeks. The eccentric cycling increased eccentric strength and induced hypertrophy–even when conducted in concurrence with conventional aerobic endurance training–in trained amateur cyclists. Still, the eccentric cycling did not improve any physiological measurements or cycling performance tests, encompassing short sprints and a 20-min time trial test. On the contrary, eccentric cycling may partly prevent improvements in cycling performance, unlike concentric cycling training. Nonetheless, the present observations were obtained immediately after the training intervention and future studies should ascertain that positive effects were not missed because of delayed adaptations.

## Supporting information

S1 Table Baseline values and changes (delta) within each group. (DOCX)
