# Supplementary material for: Eccentric cycling does not improve cycling performance in amateur cyclists
Source: PLoS One. 2019 Jan 2;14(1):e0208452. doi: 10.1371/journal.pone.0208452 (PMC6314583; doi:10.1371/journal.pone.0208452)
Supplement: S1 Table — (DOCX) [file pone.0208452.s001.docx]

S1 Table. Baseline values and changes (delta) within each group.

|  | **ECC**  **Mean ± SD**  **(n = 12)** | **Change pre-post***  **Mean ± SD** | **CON**  **Mean ± SD**  **(n = 11)** | **Change pre-**  **post***  **Mean ± SD** |
| --- | --- | --- | --- | --- |
| **Muscle thickness** |  |  |  |  |
| Vastus lateralis (VL; mm) | 2.7 ± 0.4 | 0.1 ± 0.1 | 2.6 ± 0.3 | 0.0 ± 0.1 |
| Rectus femoris (RF; mm) | 2.0 ± 0.3 | 0.1 ± 0.1 | 1.8 ± 0.2 | 0.0 ± 0.1 |
| Mean of RF and VL (mm) | 2.3 ± 0.3 | 0.1 ± 0.1 | 2.2 ± 0.2 | 0.0 ± 0.1 |
| **Strength** |  |  |  |  |
| Eccentric peak torque at 60°⋅s^-1^ (Nm) | 241 ± 43 | 29.2 ± 15.8 | 265 ± 52 | 4.7 ± 16.6 |
| Eccentric work at 60°⋅s^-1^ (J) | 317 ± 72 | 32 ± 30 | 317 ± 66 | 24 ± 16 |
| Eccentric power at 60°⋅s^-1^ (W) | 150 ± 28 | 14.8 ± 21.7 | 160 ± 28 | 1.7 ± 8.3 |
| Eccentric angle at peak torque at 60°⋅s^-1^ (°) | 79.8 ± 11.8 | -0.4 ± 5.7 | 72.9 ± 11.4 | 2.3 ± 6.6 |
| Concentric peak torque at 60°⋅s^-1^ (Nm) | 221 ± 37 | -15 ± 19 | 229 ± 33 | -9 ± 14 |
| Concentric work at 60°⋅s^-1^ (J) | 278 ± 51 | 4 ± 28 | 280 ± 60 | 36 ± 9 |
| Concentric power at 60°⋅s^-1^ (W) | 146 ± 25 | -20 ± 13 | 154 ± 23 | 34 ± 7 |
| Concentric angle at peak torque (°) | 63.4 ± 8.1 | -0.4 ± 6.2 | 64.1 ± 7.1 | 0.8 ± 2.9 |
| Isometric peak torque at 60° (Nm) | 242 ± 41 | 4 ± 32 | 263 ± 38 | -4 ± 22 |
| **Performance test** |  |  |  |  |
| 6-sec sprint mean power (W) | 1276 ± 102 | -27 ± 95 | 1251 ± 84 | -40 ± 35 |
| 30-sec sprint mean power (W) | 776 ± 62 | 1 ± 21 | 761 ± 52 | 7 ± 26 |
| 20-min time trial (W) | 268 ± 32 | 8 ± 13 | 260 ± 42 | 15 ± 14 |
| 20-min time trial (W⋅kg^-1^) | 3.6 ± 0.6 | 0.1 ± 0.1 | 3.4 ± 0.7 | 0.2 ± 0.2 |
| 20-min time trial average lactate (mmol⋅L^-1^) | 8.5 ± 1.7 | 4.2 ± 1.4 | 6.6 ± 1.5 | 2.3 ± 2.2 |
| **Endurance determinants** |  |  |  |  |
| VO_2max_ (ml) | 4668 ± 616 | 154 ± 213 | 4796 ± 518 | 244 ± 193 |
| VO_2max_ (ml⋅kg^-1^) | 62.1 ± 10.1 | 2.4 ± 3.1 | 62.0 ± 9.4 | 2.6 ± 2.6 |
| W_max_ (W) | 406 ± 54 | 7 ± 18 | 383 ± 36 | 20 ± 16 |
| 4 mmol⋅L^-1^ lactate threshold (W⋅kg^-1^) | 3.3 ± 0.7 | 0.1 ± 0.2 | 3.0 ± 0.8 | 0.2 ± 0.2 |
| Cycling economy (W⋅ml^-1^) | 16.3 ± 0.9 | 0.2 ± 1.1 | 16.9 ± 0.8 | 0.1 ± 0.4 |
| **Pedaling characteristics** |  |  |  |  |
| Pedaling peak torque (N) | 69.5 ± 7.2 | 0.5 ± 6.6 | 69.5 ± 9.8 | 4.3 ± 9.2 |
| Pedaling efficiency (%) | 86.4 ± 3.1 | 1.2 ± 2.9 | 80.6 ± 8.1 | 2.3 ± 4.0 |
| Pedaling average angle (°) | 90.2 ± 5.6 | -1.4 ± 4.0 | 90.3 ± 5.2 | 0.8 ± 3.5 |
| Pedaling min torque (N) | -9.5 ± 1.3 | 0.7 ± 1.6 | -12.8 ± 3.9 | 0.5 ± 2.9 |
| Pedaling cadence (RPM) | 94.4 ± 5.6 | -2.2 ± 3.8 | 91.0 ± 4.8 | -2.5 ± 4.3 |

*Change scores are raw-data adjusted for baseline value.

CON: Concentric cycling; ECC: Eccentric cycling; RPM: Revolutions per minute.
